# Effects of stent generation on clinical outcomes after acute myocardial infarction compared between prediabetes and diabetes patients

**DOI:** 10.1038/s41598-021-88593-x

**Published:** 2021-04-30

**Authors:** Yong Hoon Kim, Ae-Young Her, Myung Ho Jeong, Byeong-Keuk Kim, Sung-Jin Hong, Seunghwan Kim, Chul-Min Ahn, Jung-Sun Kim, Young-Guk Ko, Donghoon Choi, Myeong-Ki Hong, Yangsoo Jang

**Affiliations:** 1 Division of Cardiology, Department of Internal Medicine, Kangwon National University School of Medicine, Chuncheon, Republic of Korea; 2 Chonnam National University Hospital, Gwangju, Republic of Korea; 3 Division of Cardiology, Severance Cardiovascular Hospital, Yonsei University College of Medicine, Seoul, Republic of Korea; 4 Division of Cardiology, Inje University College of Medicine, Haeundae Paik Hospital, Busan, Republic of Korea

**Keywords:** Cardiology, Endocrinology

## Abstract

We investigated the effects of stent generation on 2-year clinical outcomes between prediabetes and diabetes patients after acute myocardial infarction (AMI). A total of 13,895 AMI patients were classified into normoglycemia (group A: 3673), prediabetes (group B: 5205), and diabetes (group C: 5017). Thereafter, all three groups were further divided into first-generation (1G)-drug-eluting stent (DES) and second-generation (2G)-DES groups. Patient-oriented composite outcomes (POCOs) defined as all-cause death, recurrent myocardial infarction (Re-MI), and any repeat revascularization were the primary outcome. Stent thrombosis (ST) was the secondary outcome. In both prediabetes and diabetes groups, the cumulative incidences of POCOs, any repeat revascularization, and ST were higher in the 1G-DES than that in the 2G-DES. In the diabetes group, all-cause death and cardiac death rates were higher in the 1G-DES than that in the 2G-DES. In both stent generations, the cumulative incidence of POCOs was similar between the prediabetes and diabetes groups. However, in the 2G-DES group, the cumulative incidences of Re-MI and all-cause death or MI were significantly higher in the diabetes group than that in the prediabetes group. To conclude, 2G-DES was more effective than 1G-DES in reducing the primary and secondary outcomes for both prediabetes and diabetes groups.

## Introduction

Diabetes mellitus (DM, diabetes) is regarded as a “coronary artery disease (CAD) risk equivalent”^1^, conferring an approximately twofold increased risk of acute myocardial infarction (AMI)^2^. Moreover, almost two thirds of those presenting with CAD have either diabetes or prediabetes^2^. Coronary vessels in patients with diabetes usually present extensive atherosclerosis with a larger number of significant stenosis, longer lesions, and more diffuse disease^3,4^. Therefore, despite advances in interventional skill, devices, and antiplatelet agents, outcomes of coronary revascularization in patients with diabetes have been poorer than those without^5,6^. Percutaneous coronary intervention (PCI) in patients with diabetes is associated with increased incidence of restenosis, repeat revascularization, stent thrombosis (ST), and all-cause mortality than those without^3,4^. Drug-eluting stents (DES) reduce the risk of restenosis as compared with bare-metal stents (BMS). However, ST remains a major concern after the implantation of first-generation (1G)-DES in patients with diabetes^7^. Relative superiority between the 1G- and 2G-DESs in patients with diabetes remains controversial^8–11^. Although recent reports revealed that prediabetes is an intergrade between normoglycemia and diabetes^12–14^, PCI patients with prediabetes were prone to experience adverse clinical events. Individuals with prediabetes are important and common patients who visit interventional cardiologists. However, the main treatment strategies for hyperglycemia are focused on the patients with diabetes rather those with prediabetes^15^. Moreover, studies regarding the effects of the 1G-DES and 2G-DES on clinical outcomes between prediabetes and diabetes patients after AMI were limited. To better understand the characteristic of prediabetes, we compared the 2-year clinical outcomes of the 1G-DES and 2G-DES under two different glycemic states (prediabetes and diabetes).

## Results

### Baseline characteristics

Table 1 summarizes the baseline clinical, laboratory, and procedural characteristics of the study population. The study population consisted of patients who had a relatively well-preserved left ventricular ejection fraction (LVEF; mean: 52.1 ± 11.4%). The mean value of high-sensitivity C-reactive protein (hs-CRP) and number of patients who received clopidogrel and cilostazole as the discharge medications were significantly higher in 1G-DES group than in 2G-DES group in all three different glycemic groups. In contrast, the number of patients requiring cardiopulmonary resuscitation (CPR) on admission; number of patients who received PCI within 24 h; number of patients who received aspirin, ticagrelor, prasugrel, beta-blockers, and lipid lowering agents as the discharge medications; American College of Cardiology/American Heart Association (ACC/AHA) type C lesion; and mean length of deployed stent were significantly higher in 2G-DES group than in 1G-DES group in all three different glycemic groups. However, the mean value of age, LVEF, body mass index, systolic blood pressure; number of ST-segment-elevation myocardial infarction (STEMI) and dyslipidemia; number of patient with previous history of PCI, coronary artery bypass graft, cerebrovascular accident, and heart failure; number of current smoker and treated vessel; mean value of serum creatinine and diameter of deployed stent; and the use of intravascular ultrasound (IVUS) were similar between the 1G-DES and 2G-DES groups in all three different glycemic groups.

**Table 1:** Baseline clinical, laboratory, and procedural characteristics.

Variables	Group A normoglycemia (n = 3673)			Group B prediabetes (n = 5205)			Group C diabetes (n = 5017)		
	Group A1 1G-DES (n = 482)	Group A2 2G-DES (n = 3191)	p value	Group B1 1G-DES (n = 767)	Group B2 2G-DES (n = 4438)	p value	Group C1 1G-DES (n = 779)	Group C2 2G-DES (n = 4238)	p value
Male, n (%)	367 (76.1)	2558 (80.2)	0.041	555 (72.4)	3276 (73.8)	0.398	539 (69.2)	3013 (71.7)	0.283
Age, years	61.6 ± 13.4	61.4 ± 13.0	0.836	64.0 ± 12.0	64.3 ± 12.4	0.488	63.4 ± 11.6	63.3 ± 11.6	0.823
LVEF, %	53.4 ± 11.9	52.9 ± 10.7	0.306	52.6 ± 12.4	52.3 ± 11.2	0.578	51.3 ± 12.1	51.2 ± 11.6	0.809
BMI, kg/m^2^	23.9 ± 2.8	23.8 ± 3.1	0.556	24.1 ± 3.1	24.1 ± 3.3	0.892	24.5 ± 3.1	24.5 ± 3.2	0.699
SBP, mmHg	129.9 ± 26.9	131.2 ± 27.8	0.354	130.4 ± 27.9	129.5 ± 27.7	0.421	130.5 ± 25.9	131.6 ± 28.1	0.319
DBP, mmHg	80.3 ± 16.7	80.6 ± 16.7	0.791	80.2 ± 16.1	78.7 ± 16.3	0.017	78.9 ± 15.5	79.1 ± 16.4	0.825
Cardiogenic shock, n (%)	16 (3.3)	126 (3.9)	0.612	41 (5.3)	203 (4.6)	0.351	18 (2.3)	190 (4.5)	0.004
CPR on admission, n (%)	6 (1.2)	154 (4.8)	< 0.001	19 (2.5)	217 (4.9)	0.002	10 (1.3)	167 (3.9)	< 0.001
STEMI, n (%)	283 (58.7)	1894 (59.4)	0.790	437 (57.0)	2576 (58.0)	0.580	438 (56.2)	2276 (53.7)	0.194
Primary PCI, n (%)	264 (93.3)	1825 (96.4)	0.014	407 (93.1)	2477 (96.2)	0.004	415/ (94.7)	2177/ (95.7)	0.404
NSTEMI, n (%)	199 (41.3)	1298 (40.7)	0.790	330 (43.0)	1862 (42.0)	0.580	341 (43.8)	1962 (46.3)	0.194
PCI within 24 h	144 (72.4)	1146 (88.3)	< 0.001	255 (77.3)	1592/ (85.5)	< 0.001	247/ (72.4)	1654/ (84.3)	< 0.001
Hypertension, n (%)	228 (47.3)	1333 (41.8)	0.022	393 (51.2)	2188 (49.3)	0.322	432 (55.5)	2427 (57.3)	0.348
Dyslipidemia, n (%)	36 (7.5)	271 (8.5)	0.481	17 (10.0)	524 (11.8)	0.178	117 (15.0)	623 (14.7)	0.826
Previous MI, n (%)	14 (2.9)	98 (3.1)	0.843	20 (2.6)	137 (3.1)	0.567	21 (2.7)	196 (4.6)	0.013
Previous PCI, n (%)	17 (3.5)	135 (4.2)	0.540	42 (5.5)	239 (5.4)	0.931	49 (6.3)	314 (7.4)	0.292
Previous CABG, n (%)	0 (0.0)	9 (0.3)	0.616	3 (0.4)	13 (0.3)	0.720	4 (0.5)	30 (0.7)	0.811
Previous CVA, n (%)	18 (3.7)	153 (4.8)	0.354	47 (6.1)	268 (6.0)	0.935	49 (6.3)	311 (7.3)	0.326
Previous HF, n (%)	2 (0.4)	18 (0.6)	0.678	8 (1.0)	50 (1.1)	0.839	18 (2.3)	62 (1.5)	0.087
Current smokers, n (%)	233 (48.3)	1441 (45.2)	0.191	309 (40.3)	1943 (43.8)	0.076	317 (40.7)	1731 (40.8)	0.968
Peak CK-MB, mg/dL	128.9 ± 149.4	139.0 ± 203.7	0.189	126.0 ± 204.3	138.3 ± 197.0	0.119	102.6 ± 152.9	106.3 ± 143.3	0.534
Peak troponin-I, ng/mL	39.5 ± 54.9	48.3 ± 75.2	0.002	40.7 ± 80.7	46.7 ± 107.5	0.072	37.7 ± 63.4	48.6 ± 94.8	0.001
NT-ProBNP, pg/mL	2307.6 ± 4254.5	1879.2 ± 3457.2	0.035	2194.5 ± 4071.7	2070.7 ± 3721.1	0.431	2549.2 ± 4658.0	2414.0 ± 5379.2	0.468
Hs-CRP, mg/dL	15.4 ± 83.9	7.9 ± 28.5	< 0.001	12.8 ± 35.4	9.8 ± 46.2	0.038	16.3 ± 62.6	11.5 ± 43.7	0.039
Serum creatinine, mg/L	1.08 ± 0.96	1.03 ± 0.99	0.268	1.12 ± 1.02	1.11 ± 1.50	0.926	1.26 ± 2.50	1.18 ± 1.66	0.352
Total cholesterol, mg/dL	183.4 ± 40.2	180.6 ± 40.8	0.161	187.7 ± 43.2	186.0 ± 44.1	0.332	186.3 ± 47.6	181.1 ± 48.4	0.005
Triglyceride, mg/L	116.4 ± 75.8	118.3 ± 88.0	0.625	118.4 ± 74.4	131.5 ± 101.1	< 0.001	153.4 ± 124.4	157.1 ± 136.7	0.459
HDL cholesterol, mg/L	44.7 ± 12.7	44.4 ± 15.2	0.616	44.6 ± 12.7	43.4 ± 15.1	0.022	43.3 ± 22.9	41.8 ± 14.2	0.090
LDL cholesterol, mg/L	117.2 ± 34.8	114.6 ± 36.0	0.120	120.4 ± 37.4	118.8 ± 45.0	0.304	116.8 ± 42.4	112.0 ± 38.5	0.003
Diabetes management									
Diet, n (%)							53 (6.8)	306 (7.2)	0.762
Oral agent, n (%)							469 (60.2)	2488 (58.7)	0.435
Insulin, n (%)							49 (6.3)	252 (5.9)	0.710
Untreated, n (%)							208 (26.7)	1192 (28.1)	0.413
**Discharge medications**									
Aspirin, n (%)	454 (94.2)	3092 (96.9)	0.002	724 (94.4)	4276 (96.3)	< 0.001	726 (93.2)	4072 (96.1)	< 0.001
Clopidogrel, n (%)	477 (99.0)	2573 (80.6)	< 0.001	746 (97.3)	3810 (85.8)	< 0.001	757 (97.2)	3624 (85.5)	< 0.001
Ticagrelor, n (%)	1 (0.2)	382 (12.0)	< 0.001	4 (0.5)	382 (8.6)	< 0.001	8 (1.0)	328 (7.7)	< 0.001
Prasugrel, n (%)	0 (0.0)	198 (6.2)	< 0.001	4 (0.5)	203 (4.6)	< 0.001	2 (0.3)	204 (4.8)	< 0.001
Cilostazole, n (%)	137 (28.4)	449 (14.1)	< 0.001	223 (29.1)	848 (19.1)	< 0.001	231 (29.7)	830 (19.6)	< 0.001
Beta-blockers, n (%)	380 (78.8)	2651 (83.1)	0.022	608 (79.3)	3680 (82.9)	< 0.001	594 (76.3)	3522 (83.1)	< 0.001
ACEIs, n (%)	308 (63.9)	1843 (57.8)	0.011	444 (57.9)	2404 (54.2)	0.012	438 (56.2)	2176 (51.3)	0.012
ARBs, n (%)	91 (18.9)	765 (24.0)	0.014	185 (24.1)	1133 (25.5)	0.004	189 (24.3)	1240 (29.3)	0.004
CCBs, n (%)	37 (7.7)	181 (5.7)	0.083	55 (7.2)	245 (5.5)	0.248	68 (8.7)	319 (7.5)	0.248
Lipid lowering agents	393 (81.5)	2876 (90.1)	< 0.001	618 (80.6)	3937 (88.7)	< 0.001	601 (77.2)	3645 (86.0)	< 0.001
**IRA**									
Left main, n (%)	7 (1.5)	54 (1.7)	0.849	19 (2.5)	77 (1.7)	0.670	16 (2.1)	79 (1.9)	0.670
LAD, n (%)	257 (53.3)	1603 (50.2)	0.207	373 (48.6)	2179 (49.1)	0.275	377 (48.4)	1961 (46.3)	0.275
LCx, n (%)	81 (16.8)	523 (16.4)	0.819	136 (17.7)	728 (16.4)	0.174	147 (18.9)	715 (16.9)	0.174
RCA, n (%)	136 (28.2)	1010 (31.7)	0.140	239 (31.2)	1454 (32.8)	0.024	238 (30.6)	1474 (34.8)	0.024
**Treated vessel**									
Left main, n (%)	17 (3.5)	84 (2.6)	0.293	27 (3.5)	129 (2.9)	0.237	30 (3.9)	129 (3.0)	0.237
LAD, n (%)	291 (60.4)	1883 (59.0)	0.570	460 (60.0)	2591 (58.4)	0.994	458 (58.8)	2491 (58.8)	0.994
LCx, n (%)	122 (25.3)	786 (24.6)	0.747	217 (28.3)	1144 (25.8)	0.985	219 (28.1)	1190 (28.1)	0.985
RCA, n (%)	169 (35.1)	1182 (37.0)	0.418	292 (38.1)	1744 (39.3)	0.252	313 (40.2)	1799 (42.4)	0.252
**ACC/AHA lesion type**									
Type B1, n (%)	82 (17.0)	424 (13.3)	0.027	120 (15.6)	597 (13.5)	0.009	124 (15.9)	530 (12.5)	0.009
Type B2, n (%)	153 (31.7)	1064 (33.3)	0.500	248 (32.3)	1425 (32.1)	0.180	231 (29.7)	1362 (32.1)	0.180
Type C, n (%)	168 (34.9)	1424 (44.6)	< 0.001	284 (37.0)	1957 (44.1)	< 0.001	277 (35.6)	1942 (45.8)	< 0.001
**Extent of CAD**									
1-vessel, n (%)	229 (47.5)	1744 (54.7)	0.003	327 (42.6)	2234 (50.3)	< 0.001	280 (35.9)	1807 (42.6)	< 0.001
2-vessel, n (%)	166 (34.4)	962 (30.1)	0.057	251 (32.7)	1398 (31.5)	0.847	265 (34.0)	1426 (33.6)	0.847
≥ 3-vessel, n (%)	87 (18.0)	485 (15.2)	0.121	189 (24.6)	806 (18.2)	< 0.001	234 (30.0)	1005 (23.7)	< 0.001
**DESs**									
SES, n (%)	225 (46.7)			330 (43.0)			352 (45.2)		
PES, n (%)	257 (53.3)			437 (57.0)			427 (54.8)		
ZES, n (%)		1015 (31.8)			1529 (34.5)			1478 (34.9)	
EES, n (%)		1625 (50.9)			2278 (51.3)			2194 (51.8)	
BES, n (%)		525 (16.4)			600 (13.5)			536 (12.6)	
Others, n (%)		26 (0.8)			31 (0.7)			30 (0.7)	
IVUS	119 (24.7)	682 (21.4)	0.110	185 (24.1)	1038 (23.4)	0.533	156 (20.0)	894 (21.1)	0.533
OCT	0 (0.0)	24 (0.8)	0.064	1 (0.1)	34 (0.8)	0.010	0 (0.0)	31 (0.7)	0.010
FFR	1 (0.2)	30 (0.9)	0.114	1 (0.1)	60 (1.4)	0.026	2 (0.3)	46 (1.1)	0.026
Stent diameter, mm	3.16 ± 0.42	3.16 ± 0.43	0.907	3.14 ± 0.42	3.14 ± 0.42	0.098	3.07 ± 0.39	3.10 ± 0.42	0.098
Stent length, mm	25.9 ± 7.8	27.1 ± 11.4	0.004	26.0 ± 7.2	26.9 ± 11.5	0.003	26.5 ± 7.9	27.5 ± 11.8	0.003
Number of stent	1.50 ± 0.84	1.42 ± 0.75	0.053	1.55 ± 0.84	1.48 ± 0.80	0.469	1.59 ± 0.90	1.56 ± 0.84	0.469

Values are means ± SD or numbers and percentages. The p values for continuous data were obtained from the analysis of variance. The p values for categorical data were obtained from the chi-square or Fisher’s exact test.

*PCI* percutaneous coronary intervention, *BMS* bare-metal stents, *1G* first-generation, *2G* second-generation, *DES* drug-eluting stents, *LVEF* left ventricular ejection fraction, *BMI* body mass index, *CPR* cardiopulmonary resuscitation, *MI* myocardial infarction, *CABG* coronary artery bypass graft, *CVA* cerebrovascular accidents, *HF* heart failure, *CK-MB* creatine kinase myocardial band, *NT-ProBNP* N-terminal pro-brain natriuretic peptide, *Hs-CRP* high-sensitivity-C-reactive protein, *HDL* high-density lipoprotein, *LDL* low-density lipoprotein, *ACEIs* angiotensin converting enzyme inhibitors, *ARBs* angiotensin receptor blockers, *CCBs* calcium channel blockers, *IRA* infarct-related artery, *ACC/AHA* American College of Cardiology/American Heart Association, *CAD* coronary artery disease, *SES* sirolimus-eluting stent, *PES* paclitaxel-eluting stent, *ZES* zotarolimus-eluting stent, *EES* everolimus-eluting stent, *BES* biolimus-eluting stent, *IVUS* intravascular ultrasound, *OCT* optical coherence tomography, *FFR* fractional flow reserve.

### Clinical outcomes

**Figure 1:**
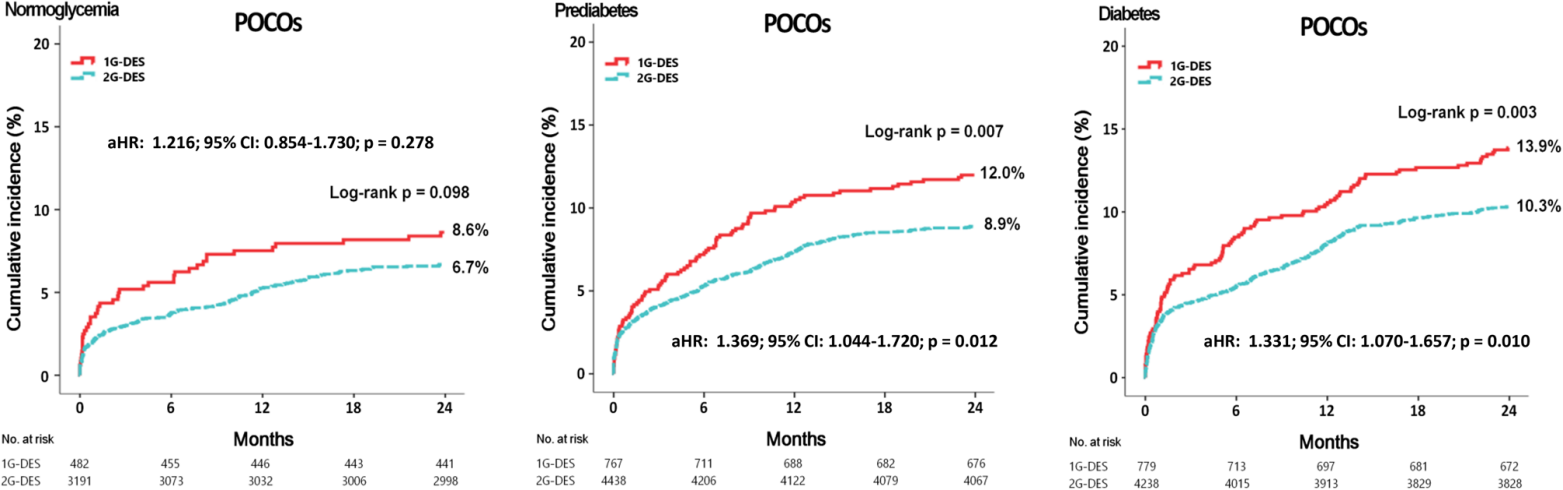
Kaplan–Meier analysis of the incidence of POCOs.

Cumulative incidences of major clinical outcomes during the 2-year follow-up period are summarized in Tables 2, 3, and Fig. 1, and Supplementary information.

**Table 2:** Clinical outcomes between 1G-DES and 2G-DES at 2 years.

Outcomes	Normoglycemia						
	Group A1 1G-DES (n = 482)	Group A2 2G-DES (n = 3191)	Log-rank	Unadjusted HR (95% CI)	p value	Adjusted^a^ HR (95% CI)	p value
POCOs	41 (8.6)	193 (6.7)	0.098	1.328 (0.948–1.861)	0.099	1.216 (0.854–1.730)	0.278
All-cause death	22 (4.6)	80 (2.7)	0.025	1.705 (1.063–2.734)	0.027	1.504 (0.914–2.474)	0.109
Cardiac death	17 (3.6)	59 (1.9)	0.027	1.825 (1.064–3.131)	0.029	1.487 (0.838–2.639)	0.176
Re-MI	8 (1.7)	42 (1.5)	0.675	1.175 (0.551–2.505)	0.676	1.180 (0.532–2.619)	0.665
All-cause death or MI	27 (5.6)	116 (4.0)	0.088	1.438 (0.945–2.187)	0.090	1.307 (0.843–2.026)	0.232
Any repeat revascularization	17 (3.6)	85 (3.1)	0.444	1.225 (0.728–2.063)	0.444	1.118 (0.645–1.938)	0.692
Stent thrombosis (probable or definite)	7 (1.5)	15 (0.5)	0.009	3.015 (1.266–7.616)	0.013	3.262 (1.226–8.678)	0.018

Outcomes	Prediabetes						
	Group B1 1G-DES (n = 767)	Group B2 2G-DES (n = 4438)	Log-rank	Unadjusted HR (95% CI)	p value	Adjusted^**b**^ HR (95% CI)	p value
POCOs	91 (12.0)	371 (8.9)	0.007	1.372 (1.091–1.726)	0.007	1.369 (1.044–1.720)	0.012
All-cause death	43 (5.6)	185 (4.4)	0.121	1.299 (0.932–1.810)	0.122	1.350 (0.939–1.845)	0.110
Cardiac death	34 (4.5)	140 (3.3)	0.098	1.370 (0.942–1.993)	0.100	1.364 (0.916–1.963)	0.132
Re-MI	19 (2.5)	80 (2.0)	0.305	1.299 (0.787–2.142)	0.306	1.293 (0.780–2.137)	0.316
All-cause death or MI	52 (6.8)	258 (6.1)	0.467	1.117 (0.829–1.505)	0.468	1.114 (0.824–1.502)	0.483
Any repeat revascularization	46 (6.3)	144 (3.6)	0.001	1.780 (1.277–2.481)	0.001	1.795 (1.280–2.518)	0.001
Stent thrombosis (probable or definite)	14 (1.8)	29 (0.7)	0.001	2.806 (1.483–5.311)	0.002	2.637 (1.370–5.077)	0.004

Outcomes	Diabetes						
	Group C1 1G-DES (n = 779)	Group C2 2G-DES (n = 4238)	Log-rank	Unadjusted HR (95% CI)	p value	Adjusted^**c**^ HR (95% CI)	p value
POCOs	107 (13.9)	410 (10.3)	0.003	1.373 (1.110–1.699)	0.003	1.331 (1.070–1.657)	0.010
All-cause death	50 (6.5)	189 (4.7)	0.037	1.390 (1.018–1.899)	0.038	1.534 (1.115–2.112)	0.009
Cardiac death	42 (5.4)	144 (3.5)	0.012	1.544 (1.095–2.178)	0.013	1.700 (1.195–2.448)	0.003
Re-MI	24 (3.2)	105 (2.8)	0.474	1.176 (0.754–1.832)	0.475	1.318 (0.833–2.085)	0.237
All-cause death or MI	64 (8.3)	296 (7.4)	0.377	1.129 (0.862–1.480)	0.378	1.290 (0.977–1.703)	0.073
Any repeat revascularization	51 (6.9)	160 (4.3)	0.001	1.661 (1.212–2.276)	0.002	1.673 (1.211–2.313)	0.002
Stent thrombosis (probable or definite)	16 (2.1)	40 (0.9)	0.007	2.189 (1.226–3.909)	0.008	2.065 (1.100–3.876)	0.024

*POCOs* patient-oriented composite outcomes defined as a composite of all-cause deaths, *Re-MI* or any repeat revascularization, *Re-MI* recurrent myocardial infarction, *LVEF* left ventricular ejection fraction, *DBP* diastolic blood pressure, *CPR* cardiopulmonary resuscitation, *PCI* percutaneous coronary intervention, *hs-CRP* high-sensitivity-C-reactive protein, *HDL* high-density lipoprotein, *LDL* low-density lipoprotein, *ACEI* angiotensin converting enzyme inhibitor, *ARB* angiotensin receptor blockers, *IRA* infarct-related artery, *RCA* right coronary artery, *ACC/AHA* American College of Cardiology/American Heart Association, *IVUS* intravascular ultrasound, *OCT* optical coherence tomography, *FFR* fractional flow reserve.

^a^Adjusted by male, age, CPR on admission, primary PCI, PCI within 24hours, hypertension, peak troponin-I, aspirin, clopidogrel, ticagrelor, prasugrel, cilostazole, beta-blocker, ACEI, ARB, lipid lowering agent, ACC/AHA type B1/C lesions, 1-vessel disease, stent length (p vales of these covariates were < 0.05 or having predictive values).

^b^Adjusted by male, age, DBP, cardiogenic shock, CPR on admission, primary PCI, PCI within 24 h, hs-CRP, triglyceride, HDL-cholesterol, aspirin, clopidogrel, ticagrelor, prasugrel, cilostazole, beta-blocker, lipid lowering agents, ACC/AHA type C lesions, 1-vessel disease, ≥ 3-vessel disease, FFR, stent length, number of stent (p vales of these covariates were < 0.05 or having predictive values).

^c^Adjusted by male, age, cardiogenic shock, CPR on admission, PCI within 24hours, previous MI, peak troponin-I, hs-CRP, total cholesterol, LDL-cholesterol, aspirin, clopidogrel, ticagrelor, prasugrel, cilostazole, beta-blocker, ACEI, ARB, lipid lowering agent, IRA (RCA), ACC/AHA type B1/C lesions, OCT, FFR, stent length (p vales of these covariates were < 0.05 or having predictive values).

**Table 3:** Two-year clinical outcomes according to the different glycemic status.

Outcomes	1G-DES						
	Group A1 normoglycemia (n = 482)	Group B1 prediabetes (n = 767)	Log-rank	Unadjusted		Adjusted^a^	
				HR (95% CI)	p value	HR (95% CI)	p value
POCOs	41 (8.6)	91 (12.0)	0.072	1.400 (1.968–2.024)	0.074	1.483 (0.985–2.232)	0.059
All-cause death	22 (4.6)	43 (5.6)	0.422	1.234 (0.738–2.062)	0.423	1.227 (0.675–2.033)	0.502
Cardiac death	17 (3.6)	34 (4.5)	0.433	1.261 (0.705–2.257)	0.435	1.455 (0.721–2.935)	0.295
Re-MI	8 (1.7)	19 (2.5)	0.334	1.498 (0.656–3.422)	0.337	1.748 (0.685–4.464)	0.243
All-cause death or MI	27 (5.6)	52 (6.8)	0.412	1.215 (0.763–1.933)	0.413	1.194 (0.707–1.919)	0.507
Any repeat revascularization	17 (3.6)	46 (6.3)	0.053	1.719 (0.986–2.999)	0.056	1.858 (1.027–3.359)	0.040
ST (probable or definite)	7 (1.5)	14 (1.8)	0.642	1.259 (0.508–3.119)	0.619	1.346 (0.511–3.547)	0.548

Outcomes	Group A1 Normoglycemia (n = 482)	Group C1 diabetes (n = 779)	Log-rank	Unadjusted		Adjusted^a^	
				HR (95% CI)	p value	HR (95% CI)	p value
POCOs	41 (8.6)	107 (13.9)	0.007	1.630 (1.137–2.336)	0.008	1.667 (1.105–2.515)	0.015
All-cause death	22 (4.6)	50 (6.5)	0.168	1.420 (0.860–2.345)	0.170	1.451 (0.797–2.639)	0.164
Cardiac death	17 (3.6)	42 (5.4)	0.130	1.540 (0.877–2.705)	0.133	1.652 (0.815–3.349)	0.129
Re-MI	8 (1.7)	24 (3.2)	0.118	1.873 (0.841–4.169)	0.124	2.500 (0.971–6.441)	0.058
All-cause death or MI	27 (5.6)	64 (8.3)	0.084	1.483 (0.946–2.325)	0.086	1.496 (0.885–2.531)	0.072
Any repeat revascularization	17 (3.6)	51 (6.9)	0.022	1.878 (1.084–3.251)	0.024	1.875 (1.029–3.215)	0.038
ST (probable or definite)	7 (1.5)	16 (2.1)	0.642	1.240 (0.500–3.071)	0.643	1.539 (0.580–4.084)	0.386

Outcomes	Group B1 Prediabetes (n = 767)	Group C1 Diabetes (n = 779)	Log-rank	Unadjusted		Adjusted^a^	
				HR (95% CI)	p value	HR (95% CI)	p value
POCOs	91 (12.0)	107 (13.9)	0.285	1.165 (0.881–1.540)	0.285	1.135 (0.836–1.535)	0.417
All-cause death	43 (5.6)	50 (6.5)	0.500	1.151 (0.765–1.730)	0.500	1.166 (0.731–1.860)	0.488
Cardiac death	34 (4.5)	42 (5.4)	0.385	1.221 (0.777–1.919)	0.386	1.137 (0.678–1.909)	0.627
Re-MI	19 (2.5)	24 (3.2)	0.462	1.253 (0.686–2.287)	0.463	1.148 (0.599–2.199)	0.678
All-cause death or MI	52 (6.8)	64 (8.3)	0.283	1.221 (0.847–1.761)	0.284	1.189 (0.788–1.757)	0.410
Any repeat revascularization	46 (6.3)	51 (6.9)	0.663	1.093 (0.733–1.627)	0.663	1.035 (0.681–1.574)	0.872
ST (probable or definite)	14 (1.8)	16 (2.1)	0.969	1.015 (0.484–2.128)	0.969	1.175 (0.551–2.507)	0.677

Outcomes	2G-DES						
	Group A2 Normoglycemia (n = 3191)	Group B2 Prediabetes (n = 4438)	Log-rank	Unadjusted		Adjusted^b^	
				HR (95% CI)	p value	HR (95% CI)	p value
POCOs	193 (6.7)	371 (8.9)	< 0.001	1.388 (1.167–1.650)	< 0.001	1.294 (1.078–1.553)	0.006
All-cause death	80 (2.7)	185 (4.4)	< 0.001	1.642 (1.263–2.134)	< 0.001	1.353 (1.021–1.793)	0.035
Cardiac death	59 (1.9)	140 (3.3)	0.001	1.693 (1.249–2.295)	0.001	1.392 (1.004–1.930)	0.047
Re-MI	42 (1.5)	80 (2.0)	0.121	1.342 (0.924–1.950)	0.122	1.288 (0.876–1.894)	0.198
All-cause death or MI	116 (4.0)	258 (6.1)	< 0.001	1.578 (1.268–1.965)	< 0.001	1.425 (1.132–1.794)	0.003
Any repeat revascularization	85 (3.1)	144 (3.6)	0.206	1.189 (0.909–1.554)	0.206	1.223 (0.923–1.619)	0.161
ST (probable or definite)	15 (0.5)	29 (0.7)	0.296	1.392 (0.746–2.597)	0.298	1.520 (0.787–2.937)	0.213

Outcomes	Group A2 Normoglycemia (n = 3191)	Group C2 Diabetes (n = 4238)	Log-rank	Unadjusted		Adjusted^b^	
				HR (95% CI)	p value	HR (95% CI)	p value
POCOs	193 (6.7)	410 (10.3)	< 0.001	1.566 (1.320–1.859)	< 0.001	1.400 (1.165–1.683)	< 0.001
All-cause death	80 (2.7)	189 (4.7)	< 0.001	1.748 (1.346–2.271)	< 0.001	1.430 (1.074–1.095)	0.014
Cardiac death	59 (1.9)	144 (3.5)	< 0.001	1.815 (1.341–2.457)	< 0.001	1.471 (1.055–2.052)	0.023
Re-MI	42 (1.5)	105 (2.8)	0.001	1.829 (1.278–2.616)	0.001	1.694 (1.161–2.472)	0.006
All-cause death or MI	116 (4.0)	296 (7.4)	< 0.001	1.885 (1.521–2.336)	< 0.001	1.684 (1.338–2.120)	< 0.001
Any repeat revascularization	85 (3.1)	160 (4.3)	0.018	1.370 (1.053–1.783)	0.019	1.362 (1.031–1.769)	0.030
ST (probable or definite)	15 (0.5)	40 (0.9)	0.018	2.012 (1.112–3.642)	0.021	2.068 (1.125–3.869)	0.014

Outcomes	Group B2 Prediabetes (n = 4438)	Group C2 Diabetes (n = 4238)	Log-rank	Unadjusted		Adjusted^b^	
				HR (95% CI)	p value	HR (95% CI)	p value
POCOs	371 (8.9)	410 (10.3)	0.046	1.153 (1.002–1.327)	0.046	1.116 (0.962–1.294)	0.148
All-cause death	185 (4.4)	189 (4.7)	0.531	1.067 (0.871–1.307)	0.531	1.109 (0.887–1.386)	0.365
Cardiac death	140 (3.3)	144 (3.5)	0.543	1.075 (0.852–1.356)	0.544	1.062 (0.822–1.334)	0.644
Re-MI	80 (2.0)	105 (2.8)	0.036	1.368 (1.022–1.829)	0.035	1.393 (1.135–2.043)	0.032
All-cause death or MI	258 (6.1)	296 (7.4)	0.033	1.197 (1.013–1.415)	0.034	1.224 (1.023–1.524)	0.029
Any repeat revascularization	144 (3.6)	160 (4.3)	0.203	1.157 (0.924–1.449)	0.204	1.088 (0.863–1.373)	0.474
ST (definite or probable)	29 (0.7)	40 (0.9)	0.129	1.445 (0.896–2.331)	0.131	1.546 (0.942–2.538)	0.085

*POCOs* patient-oriented composite outcomes defined as a composite of all-cause deaths, *Re-MI* or any repeat revascularization, *Re-MI* recurrent myocardial infarction, *LVEF* left ventricular ejection fraction, *BMI* body mass index, *SBP* systolic blood pressure, *DBP* diastolic blood pressure, *MI* myocardial infarction, *PCI* percutaneous coronary intervention, *CABG* coronary artery bypass graft, *HF* heart failure, *CVA* cerebrovascular accidents, *CK-MB* creatine kinase myocardial band, *NT-ProBNP* N-terminal pro-brain natriuretic peptide, *HDL* high-density lipoprotein, *LDL* low-density lipoprotein, *ACEIs* angiotensin converting enzyme inhibitors, *ARBs* angiotensin receptor blockers, *CCBs* calcium channel blockers, *IRA* infarct-related artery, *RCA* right coronary artery, *ACC/AHA* American College of Cardiology/American Heart Association, *FFR* fractional flow reserve.

^a^Adjusted by male, age, LVEF, BMI, cardiogenic shock, hypertension, dyslipidemia, previous HF, current smoker, CK-MB, ACEIs, 1-vessel, ≥ 3-vessel disease, triglyceride, stent diameter (p vales of these covariates were < 0.005 or having predictive values).

^b^Adjusted by male, age, LVEF, BMI, DBP, STEMI, hypertension, dyslipidemia, previous MI, previous PCI, previous CVA, CK-MB, serum creatinine, total cholesterol, triglyceride, HDL-cholesterol, LDL-cholesterol, clopidogrel, ticagrelor, cilostazole, ACEIs, ARBs, CCB, lipid lowering agents, RCA (treated vessel), 1-vessel disease, ≥ 3-vessel disease, stent diameter, number of stent (p vales of these covariates were < 0.001 or having predictive values).

### Prediabetes group

After the adjustment, the cumulative incidences of POCOs (adjusted hazard ratio [aHR]: 1.369; 95% confidence interval [CI] 1.044–1.720; p = 0.012), any repeat revascularization (aHR: 1.795; 95% CI 1.280–2.518; p = 0.001), and ST (aHR: 2.637; 95% CI 1.370–5.077; p = 0.004) were significantly higher in the 1G-DES than that in the 2G-DES group.

### Diabetes group

After the adjustment, the cumulative incidences of POCOs (aHR: 1.331; 95% CI 1.070–1.657; p = 0.010), all-cause death (aHR: 1.534; 95% CI 1.115–2.112; p = 0.009), CD (aHR: 1.700; 95% CI 1.195–2.448; p = 0.003), any repeat revascularization (aHR: 1.673; 95% CI 1.211–2.313; p = 0.002), and ST (aHR: 2.065; 95% CI 1.100–3.876; p = 0.024) were significantly higher in the 1G-DES group than that in the 2G-DES group.

### Normoglycemia group

After the adjustment, the cumulative incidences of POCOs, all-cause death, CD, Re-MI, all-cause death or MI, and any repeat revascularization were similar between the 1G-DES and 2G-DES groups. However, the cumulative incidence of ST (aHR: 3.262; 95% CI 1.226–8.678; p = 0.018) was significantly higher in the 1G-DES than that in the 2G-DES group.

### 1G-DES group

Cumulative incidences of POCOs (aHR: 1.135; 95% CI 0.836–1.535; p = 0.417) and ST (aHR: 1.175; 95% CI 0.551–2.507; p = 0.677) were similar between prediabetes and diabetes groups. The cumulative incidence of any repeat revascularization was significantly higher in the prediabetes than that in the normoglycemia group (aHR: 1.858; 95% CI 1.027–3.359; p = 0.040). Cumulative incidences of POCOs (aHR: 1.667; 95% CI 1.105–2.515; p = 0.015) and any repeat revascularization (aHR: 1.875; 95% CI 1.029–3.215; p = 0.038) were significantly higher in the diabetes than that in the normoglycemia group.

### 2G-DES group

Cumulative incidences of POCOs (aHR: 1.116; 95% CI 0.962–1.294; p = 0.148) and ST (aHR: 1.546; 95% CI 0.942–2.538; p = 0.085) were similar between prediabetes and diabetes groups. However, cumulative incidences of Re-MI (aHR: 1.393; 95% CI 1.135–2.043; p = 0.032) and all-cause death or MI (aHR: 1.224; 95% CI 1.023–1.524; p = 0.029) in the diabetes group were significantly higher than that in the prediabetes group. Cumulative incidences of POCOs (aHR: 1.294; 95% CI 1.078–1.553; p = 0.006), all-cause death (aHR: 1.353; 95% CI 1.021–1.793; p = 0.035), CD (aHR: 1.392; 95% CI 1.004–1.930; p = 0.047), and all-cause death or MI (aHR: 1.425; 95% CI 1.132–1.794; p = 0.003) were significantly higher in the prediabetes than that in the normoglycemia group. Cumulative incidences of POCOs (aHR: 1.400; 95% CI 1.165–1.683; p < 0.001), all-cause death (aHR: 1.430; 95% CI 1.074–1.095; p = 0.014), CD (aHR: 1.471; 95% CI 1.055–2.052; p = 0.023), Re-MI (aHR: 1.694; 95% CI 1.161–2.472; p = 0.006), all-cause death or MI (aHR: 1.684; 95% CI 1.338–2.120; p < 0.001), any repeat revascularization (aHR: 1.362; 95% CI 1.031–1.769; p = 0.030), and ST (aHR: 2.068; 95% CI 1.125–3.869; p = 0.014) were significantly higher in the diabetes than that in the normoglycemia group.

Table 4 shows independent predictors for POCOs and ST at the 2-year follow-up. Old age (≥ 65 years), male sex, low LVEF (< 40%), cardiogenic shock, cardiopulmonary resuscitation on admission, and multivessel disease were significant independent predictors for POCOs. Low LVEF and < 3 mm diameter of the deployed stent were independent predictors for ST in this study.

**Table 4:** Independent predictors for POCOs and stent thrombosis at 2 years.

Variables	POCOs				Stent thrombosis			
	Univariate		Multivariate		Univariate		Multivariate	
	HR (95% CI)	p value	HR (95% CI)	p value	HR (95% CI)	p value	HR (95% CI)	p value
1G-DES vs. 2G-DES	1.382 (1.200–1.592)	< 0.001	1.415 (1.226–1.633)	< 0.001	2.451 (1.652–3.636)	< 0.001	2.668 (1.786–3.988)	< 0.001
Age (≥ 65 years)	1.631 (1.455–1.828)	< 0.001	1.356 (1.196–1.537)	< 0.001	1.131 (0.787–1.624)	0.506	1.325 (0.890–1.974)	0.166
Male	1.452 (1.289–1.637)	< 0.001	1.178 (1.034–1.342)	0.014	1.151 (0.773–1.713)	0.488	1.084 (0.701–1.676)	0.717
LVEF (< 40%)	2.487 (2.186–2.831)	< 0.001	2.133 (1.870–2.432)	< 0.001	1.846 (1.188–2.870)	0.006	1.775 (1.133–2.782)	0.012
Hypertension	1.291 (1.153–1.447)	< 0.001	1.116 (0.991–1.257)	0.071	1.033 (0.721–1.480)	0.860	1.102 (0.756–1.605)	0.613
Dyslipidemia	1.051 (0.885–1.247)	0.572	1.052 (0.884–1.251)	0.568	1.502 (0.929–2.429)	0.097	1.482 (0.911–2.411)	0.113
Cardiogenic shock	1.673 (1.335–2.096)	< 0.001	1.284 (1.020–1.617)	0.033	1.400 (0.652–3.004)	0.388	1.350 (0.620–2.938)	0.450
CPR on admission	3.668 (3.079–4.370)	< 0.001	3.251 (2.714–3.894)	< 0.001	1.687 (0.823–3.457)	0.153	1.479 (0.708–3.088)	0.298
Multivessel disease	1.630 (1.449–1.833)	< 0.001	1.489 (1.321–1.678)	< 0.001	1.256 (0.873–1.808)	0.219	1.128 (0.779–1.633)	0.523
ACC/AHA type B2/C lesion	1.171 (1.023–1.341)	0.022	1.078 (0.939–1.237)	0.287	1.582 (0.979–2.558)	0.061	1.531 (0.940–2.493)	0.087
Stent diameter < 3.0 mm	1.195 (1.060–1.347)	0.004	1.098 (0.972–1.241)	0.132	2.537 (1.771–3.634)	< 0.001	2.518 (1.745–3.635)	< 0.001
Stent length ≥ 28 mm	1.202 (1.073–1.345)	0.001	1.071 (0.954–1.202)	0.247	1.335 (0.932–1.913)	0.115	1.145 (0.793–1.653)	0.470

*1G* first-generation, *2G* second-generation, *DES* drug-eluting stent, *POCOs* patient-oriented composite outcomes, *HR* hazard ratio, *LVEF* left ventricular ejection fraction, *CPR* cardiopulmonary resuscitation, *ACC/AHA* American College of Cardiology/American Heart Association.

## Discussion

The primary findings of this study are as follows: (1) in both prediabetes and diabetes groups, the cumulative incidences of POCOs, any repeat revascularization, and ST were higher in the 1G-DES than that in the 2G-DES; (2) in the diabetes group, the cumulative incidences of all-cause death and CD were higher in the 1G-DES than that in the 2G-DES; (3) in the normoglycemia group, the cumulative incidence of ST was higher in the 1G-DES than that in the 2G-DES; and (4) in two different stent generations, the cumulative incidence of POCOs was similar between the prediabetes and diabetes groups. However, in the 2G-DES group, the cumulative incidences of Re-MI and all-cause death or MI were higher in the diabetes group than that in the prediabetes group.

Hyperglycemia, elevated free fatty acid level, and increased amount of circulating glucosylated serum products can accelerate atherosclerosis and vascular injury in patients with diabetes by inducing endothelial dysfunction and vascular inflammation^16^. Although previous reports demonstrated that the higher rates of repeat revascularizations and mortality after PCI in patients with diabetes are caused by restenosis and disease progression^4–6^, comparative clinical outcomes between prediabetes and diabetes were not well illuminated especially, between 1G-DES and 2G-DES. Some recent reports showed that prediabetes is associated with poorer clinical outcomes including cardiovascular mortality and patients with prediabetes and diabetes have similar higher risk profiles compared with normoglycemia^13,14,17^.

Although DES improved outcomes of high-risk patients by reducing the rate of restenosis as compared with BMS^18,19^, ST remains a major concern after the DES implantation, especially in diabetes^4^. Relative superiority between the 1G-DES and 2G-DES in patients with AMI and diabetes remains controversial, and most previous studies were not performed during the prediabetes stage^10,20,21^. In our study, the cumulative incidence of POCOs was significantly higher in the 1G-DES than that in the 2G-DES in both prediabetes and diabetes groups. Moreover, in two different stent generations, the cumulative incidence of POCOs was similar between the prediabetes and diabetes groups (Table 3). In a substudy of the multicenter BIO-RESORT (BIOdegradable Polymer and DuRable Polymer Drug-eluting Stents in an All COmeRs PopulaTion) trial^13^, comparative clinical outcomes were similar between prediabetes and diabetes (11.1% vs. 10.5%). Von Birgelen et al.^22^ reported the results of the BIO-RESORT Silent Diabetes Study. In their study, the cumulative incidence of major adverse cardiac events was different between patients with prediabetes (5.5%) and normoglycemia (3.0%) (Log-rank, p = 0.07). As mentioned, despite the combination of new platforms, more biocompatible polymers were utilized in 2G-DES, the relative superiority between 1G- and 2G-DESs in patients with diabetes remains controversial^8–11^. In the SPIRIT V Diabetic Study^10^, everolimus-eluting stent (EES) was superior to paclitaxel-eluting stent (PES) for in-stent late loss at 9 months. The composite death, MI, and TVR rates were the same in the two groups at 1 year. Bavishi et al.^9^ reported that EES showed significantly lower incidence rates of MACEs by 18% and ST by 46% as compared with the 1G-DES. Moreover, the EES showed a trend toward reduced incidence rates of target lesion revascularization (TLR) and TVR (p = 0.05). In this study, based on the cumulative incidences of POCOs, any revascularization rate was significantly higher in the 1G-DES than that in the 2G-DES group in both prediabetes and diabetes group. Therefore, the major clinical outcomes of our study could reflect the meta-analysis results of Bavishi et al.’s study^9^.

The overall rate of ST was also higher in the 1G-DES than in the 2G-DES in all three different glycemic groups (prediabetes [1.8% vs. 0.7%, log-rang p = 0.001], diabetes [2.1% vs. 0.9%, log-rank p = 0.007], and normoglycemia [1.5% vs. 0.5%, log-rank p = 0.009]). With regard to prediabetes, follow-up data on the comparative long-term effects of 1G-DES and 2G-DES implantation were limited. According to Bavishi et al.’s report^9^, EES reduced the incidence of ST by 46% (RR: 0.54, 95% CI 0.35–0.82) as compared with the 1G-DES in patients with diabetes. The cumulative incidence of ST also higher in the 1G-DES than that in the 2G-DES in patients with normoglycemia. Our result is consistent with the result of Nakatsuma et al. study^23^. This low cumulative incidence of 2G-DES may be related with relatively thin stent struts (50–90 μm) and improved ability for deliverability while maintaining an adequate radial strength^24^ and more compatible and thromboresistant than those in the 1G-DES^25^. However, in our study, the occurrence of ST was high within 6 months after index PCI (Supplementary Fig. 1). Therefore, we cannot completely exclude the possibility that ST was associated with PCI procedure^26^. Even though IVUS-guided^27^ or functional flow reserve (FFR)-guided PCI^28^ could reduce MACE rate, the number of PCI base on these intracoronary image- or functional study-based PCI were less than 30% in our study. Unfortunately, currently under the Korea’s health insurance system, the reimbursement program for the use of IVUS, optical coherence of tomography, or fractional flow reserve during the PCI is very limited or absent^29^.

Interestingly, comparative clinical outcomes of the two different stent generations according to glycemic status showed some different results (Table 3). Different clinical outcomes among three different glycemic states (normoglycemia, prediabetes, and diabetes) were more prominent in the 2G-DES rather 1G-DES. According to advances in interventional skill, devices, and antiplatelet agents^5,6^, 2G-DES showed decreased incidences of all-cause death (aHR: 1.534; 95% CI 1.115–2.112; p = 0.009) and CD (aHR: 1.700; 95% CI 1.195–2.448; p = 0.003) compared with 1G-DES in diabetes group after adjustment (Table 2). Bavishi et al.^9^ showed that there was a trend towards reduction in all-cause mortality with zotarolimus compared to 1G-DES (6.3% vs. 7.2%, relative risk: 0.74; 95% CI 0.55–1.00; p = 0.05) in their meta-analysis. However, the cumulative incidences of all clinical outcomes were significantly higher in the diabetes than that in the normoglycemia group. These results may reflect hazardous effects of diabetes are sustained even in the era of 2G-DES.

In our study, in the 1G-DES, the primary and secondary end-points were similar between the prediabetes and diabetes groups. However, in the 2G-DES, the cumulative incidences of Re-MI (aHR: 1.393; 95% CI 1.135-2.043; p = 0.032) and all-cause death or MI (aHR: 1.224; 95% CI 1.023–1.524; p = 0.029) were significantly higher in the diabetes group than that in the prediabetes group. Although the precise mechanisms of the higher incidence of Re-MI in diabetes group are not fully known, one report^30^ suggested that the association between diabetes and Re-MI may be related with a direct effect of diabetes. According to recent reports^14,31^, the cumulative incidence of Re-MI of the diabetes group was significantly higher than that of the prediabetes group (aHR: 1.884; 95% CI 1.201–2.954; p = 0.006 or aHR: 1.660; 95% CI 1.000–2.755; p = 0.020).

More than 50 high-volume university or community hospitals in South Korea participated in this study. The limited reports on the impact of stent generation on long-term clinical outcomes in AMI patients with prediabetes or diabetes were the motivation for the current study. Thus, we believe that our study may provide significant information to interventional cardiologists who perform PCI in patients with AMI with prediabetes or diabetes.

This study has several limitations. First, because the study population was obtained from the Korea AMI registry data, some data might be under-reported and/or missed. Second, it is necessary for diagnosing diabetes to check an HbA1c level ≥ 6.5%, FPG ≥ 126 mg/dL (7.0 mmol/L), and/or RPG ≥ 200 mg/dL (11.1 mmol/L) by repeat testing. If first glycemic status was diabetes and second was prediabetes, or first glycemic status was prediabetes and second was normoglycemia, and this is particularly important in patients with AMI, because such patients reveal hyperglycemia in acute phase. However, in this study, the definitions of prediabetes and diabetes were not based on repeat testing. Moreover, considering the limitation of HbA1c, any other diagnostic tests for diabetes including oral glucose tolerance test are needed for a finer classification. However, detailed information on this variable was not included in the KAMIR. Hence, the results of this study can be altered based on other diagnostic tests and which directly influences the assignment of participants, and this factor may have served as an important bias in this study^32^. Third, the duration and types of antidiabetic treatment are major determinants after PCI in patents with prediabetes or diabetes. However, this study was conducted based on discharge medications, and owing to limitation of registry study, we did not precisely know the adherence or non-adherence of enrolled patients to antidiabetic drugs during the follow-up period. Therefore, this may act as an important bias in this study. Fourth, 2G-DES consisted of durable-polymer-coated DES and biodegradable-polymer-coated DES. The number of biodegradable-polymer DES (BES) was highest in the normoglycemia group (prediabetes: 600/4438 (13.5%); diabetes: 536/4238 (12.6%); normoglycemia: 525/3191 (16.5%); p < 0.001) (Table 1). Although this number was not significantly different between prediabetes and diabetes (p = 0.226), this division may be not reasonable and the composition of 2G-DES could be changed according to other types of utilized newer-generation DES. Therefore, other types of newer-generation DES could influence the outcome of our study. Fifth, although multivariate analysis was performed to strengthen our results, variables not included in the KAMIR may have affected the study outcomes. Sixth, the 2-year follow-up period in this study was relatively short in order to determine the long-term major clinical outcomes; therefore, data from studies with longer follow-up periods are required. Seventh, this study retrospectively enrolled the patients who underwent PCI from 2005–2015. The development of stent platform, potent antiplatelet drugs, and use of intracoronary imaging and improvement of procedural skills, all these factors substantially affect the clinical outcomes. Therefore, these factors could be also important bias of this study. Finally, although 2G-DES are considered the safest in the general population, this study confirms that in a select and growing population.

In conclusion, in this study, we observed that 2G-DES was more effective than 1G-DES in reducing POCOs, any repeat revascularization and ST in both prediabetes and diabetes group. Moreover, in two different stent generations, and the cumulative incidence of POCOs was similar between the prediabetes and diabetes groups. However, further studies regarding the most advanced DES technology joined with the most advanced anti-thrombotic regimen are needed to confirm these results.

## Methods

### Study population

**Figure 2:**
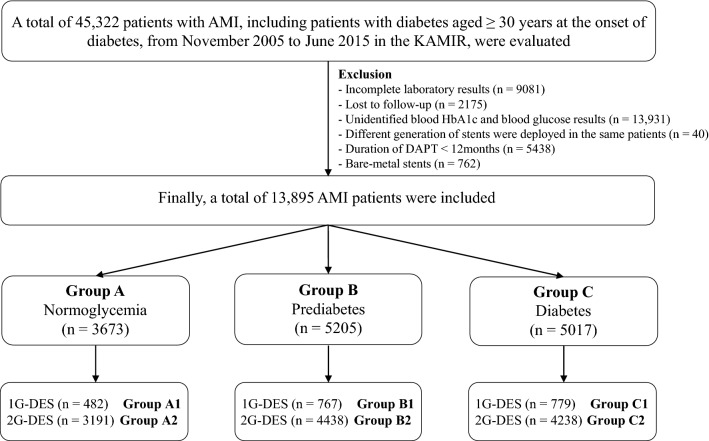
Study flow chart. AMI, acute myocardial infarction; KAMIR, Korea AMI Registry; HbA1c, hemoglobin A1c; 1G, first-generation; 2G, second-generation; DES, drug-eluting stent; DAPT, dual antiplatelet therapy.

A total of 45,322 patients with AMI who underwent successful stent implantation, including patients with DM aged ≥ 30 years at the onset of diabetes, from November 2005 to June 2015 in the KAMIR, were evaluated. Details of the registry can be found at the KAMIR website (http://www.kamir.or.kr) ^33^. Among them, patients with incomplete laboratory results (n = 9081, 20.0%), those who were lost to follow-up (n = 2175, 4.8%), those with unidentified blood hemoglobin (Hb) A1c and blood glucose level results (n = 13,931, 30.7%), those with different generations of stents were deployed in the same patients (n = 40, 0.1%), those who received dual antiplatelet therapy (DAPT) less than 12 months (n = 5438, 12.0%), and those who received BMS (n = 762, 1.7%). Finally, a total of 13,895 patients with AMI who underwent successful implantation were considered for inclusion. Patients were classified into normoglycemia (group A: 3673; 26.4%), prediabetes (group B: 5205; 37.5%), and diabetes (group C: 5017; 36.1%) (Table 1). Subsequently, all three groups were further divided into 1G-DES (group A1, group B1, and group C1) and 2G-DES groups (group A2, group B2, and group C2) (Fig. 2). The study protocol was approved by the institutional review board of each participating center and the Chonnam National University Hospital Institutional Review Board ethics committee approved (approval number: CNUH-2011-172) the study protocol. The study has been performed in accordance with the ethical standards laid down in the 1975 Declaration of Helsinki. All persons gave their written informed consent prior to their inclusion in the study. All 13,895 patients completed the 2-year clinical follow-up by face-to-face interviews, phone calls, or medical chart review. All clinical events were evaluated by an independent event adjudicating committee. The event adjudication processes have been described in a previous publication of KAMIR investigators^34^.

### Percutaneous coronary intervention and medical treatment

Before PCI, all patients were administered loading doses of aspirin 200–300 mg and clopidogrel 300–600 mg; alternatively, ticagrelor 180 mg or prasugrel 60 mg was administered. PCI was performed via the femoral or radial approach after an intravenous bolus dose of heparin (50–100 U/kg) to achieve an activated clotting time of > 250 s. DAPT (a combination of aspirin 100 mg/day with clopidogrel 75 mg/day or ticagrelor 90 mg twice daily or prasugrel 5–10 mg/day) was recommended for > 12 months for patients who underwent PCI. Triple antiplatelet therapy (TAPT: cilostazol 100 mg twice daily in addition to DAPT) was left to the discretion of the individual operators. Diagnostic coronary angiography and PCI were performed using standard guideline^35^.

### Study definitions and clinical outcomes

Glycemic status was determined based on medical history and glycated hemoglobin (HbA1c), fasting plasma glucose (FPG), and random plasma glucose (RPG) levels at the index hospitalization. According to the American Diabetes Association clinical practice recommendation^32^, prediabetes was defined as an HbA1c of 5.7–6.4% and an FPG of 100–125 mg/dL (5.6–6.9 mmol/L). Diabetes was categorized as either known diabetes defined as ongoing medical treatment for diabetes (insulin or antidiabetics), or newly diagnosed diabetes, defined as an HbA1c level ≥ 6.5%, FPG ≥ 126 mg/dL (7.0 mmol/L), and/or RPG ≥ 200 mg/dL (11.1 mmol/L). If the admission electrocardiogram of patients who complained of chest pain showed ST-segment elevations in at least two contiguous leads of ≥ 2 mm (0.2 mV) in men, or ≥ 1.5 mm (0.15 mV) in women in leads V2–V3 and/or ≥ 1 mm (0.1 mV) in other contiguous chest leads or limb leads or new-onset left bundle branch block, the patients were considered to have STEMI^36^, whereas patients who did not show persistent ST-segment elevation with increased cardiac biomarkers and with appropriate clinical context were considered to have non-STEMI (NSTEMI)^37^. In cases of NSTEMI, an early invasive treatment strategy was defined as PCI within 24 h after admission^37^. A successful PCI was defined as a residual stenosis of < 30% and more than grade 3 flow in Thrombolysis In Myocardial Infarction flow for the infarct-related artery (IRA) after the procedure. The primary outcome of this study was the occurrence of POCOs, defined as all-cause death, Re-MI, or any coronary repeat revascularization^38^. The secondary outcome was definite or probable ST during the 2-year follow-up period. All-cause death was classified as CD or non-CD. Any repeat revascularization comprised target lesion revascularization, target vessel revascularization, and non-TVR. Re-MI, TLR, TVR, and non-TVR definitions have already been published previously^39,40^. The cumulative incidence of ST was defined by the current consensus^41^.

### Statistical analyses

For continuous variables, differences between the two groups were evaluated with the unpaired t-test. Additionally, differences among the three glycemic groups were evaluated using analysis of variance or the Jonckheere–Terpstra test, whereas a post-hoc analysis of the two groups was performed using the Hochberg test or Dunnett T3 test^14^; data were expressed as mean ± standard deviation. For categorical variables, intergroup differences were analyzed using chi-squared test or Fisher’s exact test, as appropriate. Data were expressed as numbers and percentages^14^. The Kaplan–Meier method was used to estimate various clinical outcomes, and the log-rank test was used to compare intergroup differences (Fig. 1 and Supplementary information). Variables with a p value of < 0.001^14^ or < 0.05^42^ in the univariate analysis and conventional risk factors of poor outcomes in the AMI population were considered potential confounding factors and were entered into the multivariate analysis. These included variables shown in Tables 2 and 3. For all analyses, two-sided values of p < 0.05 were considered statistically significant. All statistical analyses were performed using the SPSS software version 20 (IBM; Armonk, NY, USA)^14^.

## Supplementary Information

Supplementary Information

## Data Availability

Data is contained with the article or supplementary information.
