# Supplementary material for: Effects of stent generation on clinical outcomes after acute myocardial infarction compared between prediabetes and diabetes patients
Source: Sci Rep. 2021 Apr 30;11:9364. doi: 10.1038/s41598-021-88593-x (PMC8087777; doi:10.1038/s41598-021-88593-x)
Supplement: Supplementary file 1 — Supplementary Information [file 41598_2021_88593_MOESM1_ESM.pdf]

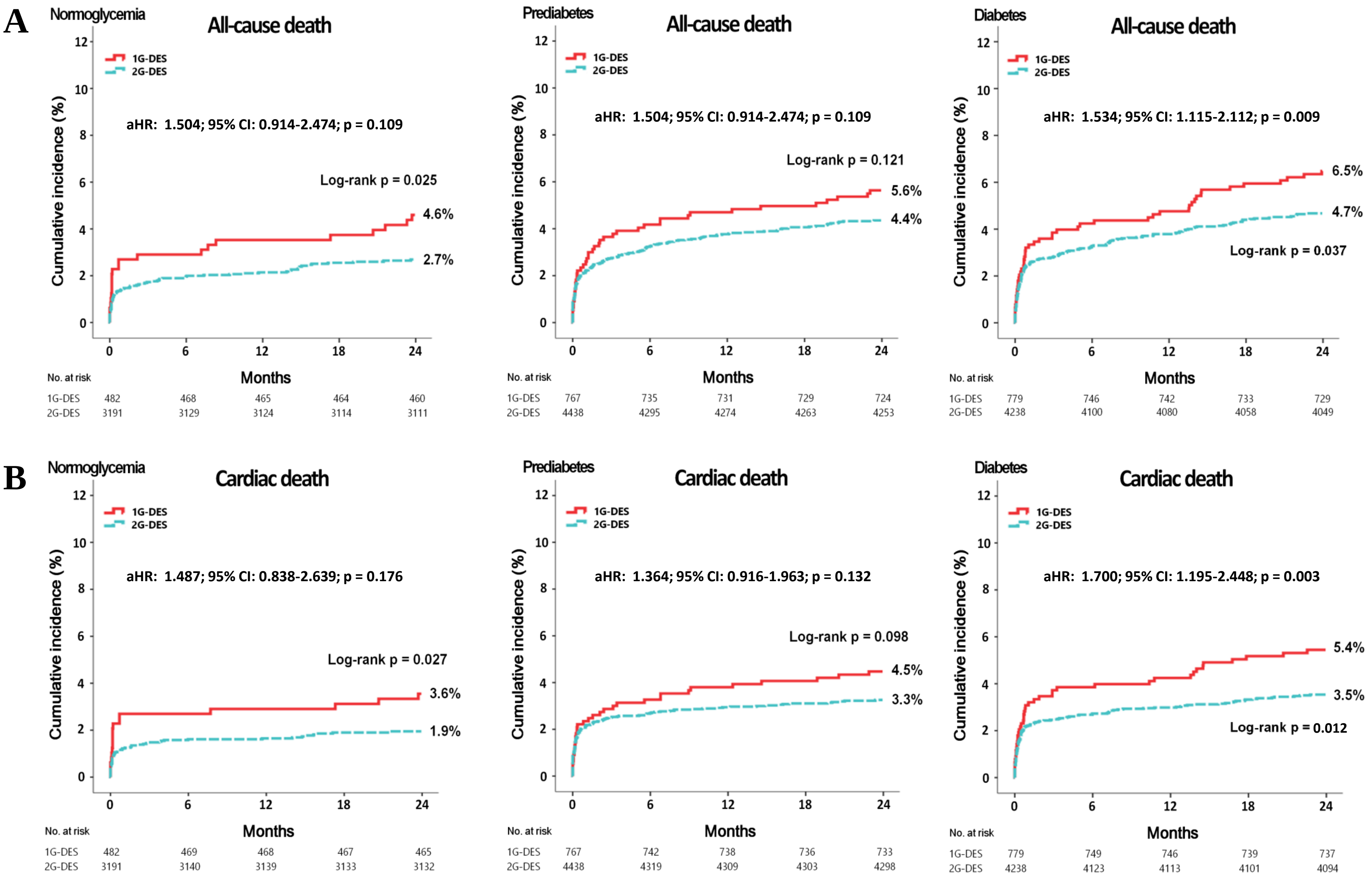

Supplementary Information 1. Kaplan-Meier analysis of the incidences of all-cause death (A), cardiac death (B), Re-MI (C), all-cause death and MI (D), any repeat revascularization (E), and stent thrombosis (F). Re-MI, recurrent myocardial infarction; 1G, first-generation; 2G, second-generation; DES, drug-eluting stent; aHR, adjusted hazard ratio.

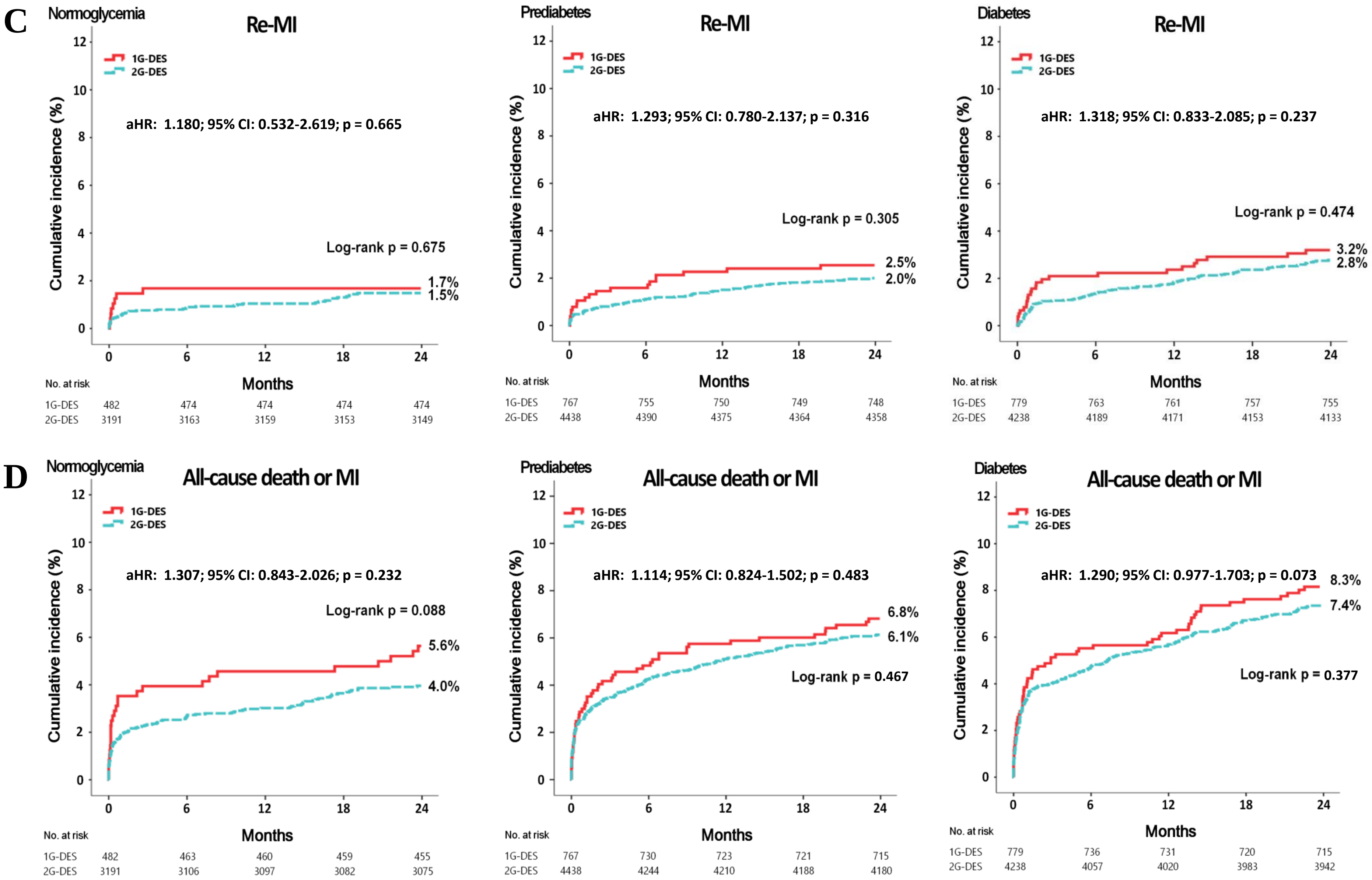
C
Normoglycemia
Re-MI
1G-DES
2G-DES
aHR: 1.180; 95% CI: 0.532-2.619; p = 0.665
Log-rank p = 0.675
1.7%
1.5%
Cumulative incidence (%)
Months
No. at risk
1G-DES 482 474 474 474 474
2G-DES 3191 3163 3159 3153 3149
Prediabetes
Re-MI
aHR: 1.293; 95% CI: 0.780-2.137; p = 0.316
Log-rank p = 0.305
2.5%
2.0%
1G-DES 767 755 750 749 748
2G-DES 4438 4390 4375 4364 4358
Diabetes
Re-MI
aHR: 1.318; 95% CI: 0.833-2.085; p = 0.237
Log-rank p = 0.474
3.2%
2.8%
1G-DES 779 763 761 757 755
2G-DES 4238 4189 4171 4153 4133
D
Normoglycemia
All-cause death or MI
aHR: 1.307; 95% CI: 0.843-2.026; p = 0.232
Log-rank p = 0.088
5.6%
4.0%
1G-DES 482 463 460 459 455
2G-DES 3191 3106 3097 3082 3075
Prediabetes
All-cause death or MI
aHR: 1.114; 95% CI: 0.824-1.502; p = 0.483
6.8%
6.1%
Log-rank p = 0.467
1G-DES 767 730 723 721 715
2G-DES 4438 4244 4210 4188 4180
Diabetes
All-cause death or MI
aHR: 1.290; 95% CI: 0.977-1.703; p = 0.073
8.3%
7.4%
Log-rank p = 0.377
1G-DES 779 736 731 720 715
2G-DES 4238 4057 4020 3983 3942

E

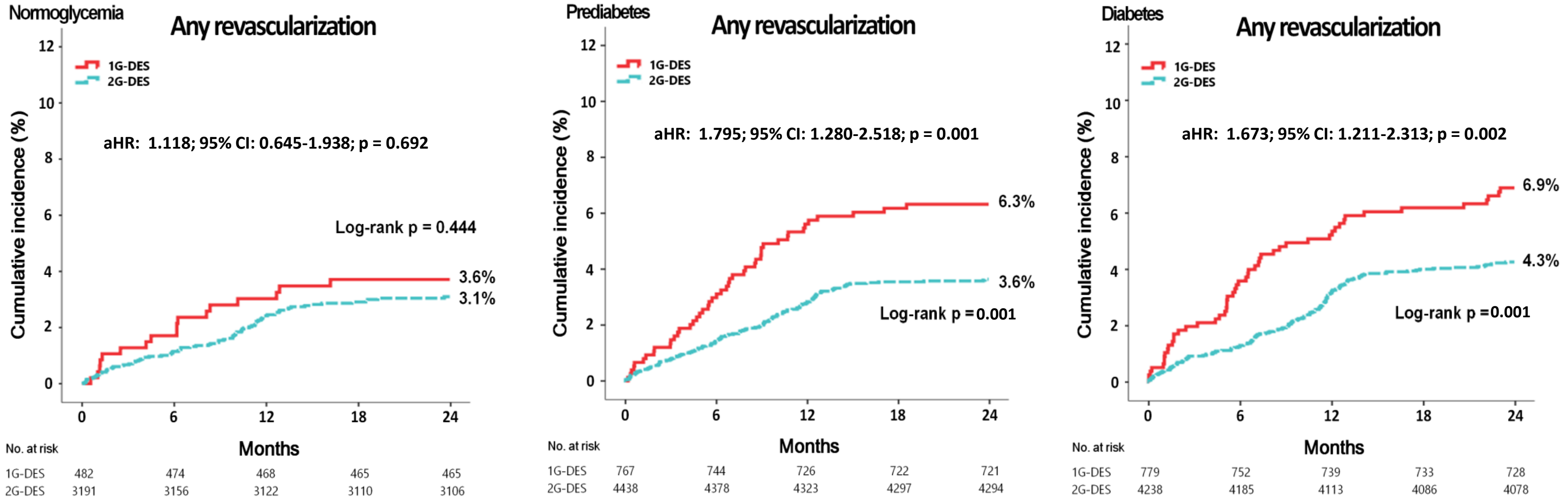
Normoglycemia
Any revascularization
1G-DES
2G-DES
aHR: 1.118; 95% CI: 0.645-1.938; p = 0.692
Log-rank p = 0.444
3.6%
3.1%
Cumulative incidence (%)
Months
No. at risk
1G-DES 482 474 468 465 465
2G-DES 3191 3156 3122 3110 3106
Prediabetes
Any revascularization
1G-DES
2G-DES
aHR: 1.795; 95% CI: 1.280-2.518; p = 0.001
6.3%
3.6%
Log-rank p = 0.001
Cumulative incidence (%)
Months
No. at risk
1G-DES 767 744 726 722 721
2G-DES 4438 4378 4323 4297 4294
Diabetes
Any revascularization
1G-DES
2G-DES
aHR: 1.673; 95% CI: 1.211-2.313; p = 0.002
6.9%
4.3%
Log-rank p =0.001
Cumulative incidence (%)
Months
No. at risk
1G-DES 779 752 739 733 728
2G-DES 4238 4185 4113 4086 4078

F

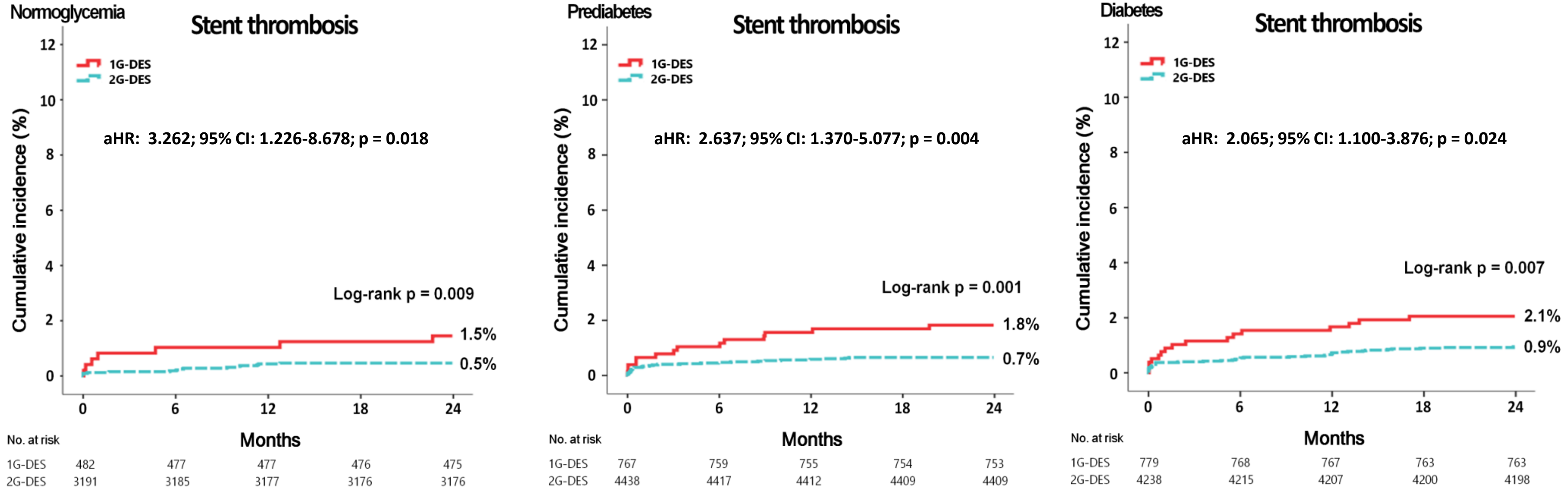
Normoglycemia
Stent thrombosis
1G-DES
2G-DES
aHR: 3.262; 95% CI: 1.226-8.678; p = 0.018
Log-rank p = 0.009
1.5%
0.5%
Cumulative incidence (%)
Months
No. at risk
1G-DES 482 477 477 476 475
2G-DES 3191 3185 3177 3176 3176
Prediabetes
Stent thrombosis
1G-DES
2G-DES
aHR: 2.637; 95% CI: 1.370-5.077; p = 0.004
Log-rank p = 0.001
1.8%
0.7%
Cumulative incidence (%)
Months
No. at risk
1G-DES 767 759 755 754 753
2G-DES 4438 4417 4412 4409 4409
Diabetes
Stent thrombosis
1G-DES
2G-DES
aHR: 2.065; 95% CI: 1.100-3.876; p = 0.024
Log-rank p = 0.007
2.1%
0.9%
Cumulative incidence (%)
Months
No. at risk
1G-DES 779 768 767 763 763
2G-DES 4238 4215 4207 4200 4198
